# The efficacy of orally administered L-carnitine in alleviating ovarian dysfunctions has laid the foundation for targeted *in vivo* use: a study employing self-control and propensity score matching

**DOI:** 10.3389/fendo.2024.1440182

**Published:** 2024-09-18

**Authors:** Wenjie Zhao, Kunkun Liu, Yuhua Zhang, Pingping Sun, Ernest Zeringue, Li Meng, Huagang Ma

**Affiliations:** ^1^ Reproductive Medicine Center, Weifang People’s Hospital, Weifang, Shandong, China; ^2^ IVF Laboratories, California IVF Fertility Center, Sacramento, CA, United States

**Keywords:** L-carnitine, propensity score matching, oocyte, normal fertilization, blastocyst formation

## Abstract

**Objective:**

This study aimed to evaluate the effectiveness of oral L-carnitine administration in patients after treatment failure to lay the groundwork for targeted *in vivo* use.

**Methods and materials:**

A total of 515 *In Vitro* Fertilization (IVF) patients undergoing subsequent cycles were included after applying exclusion criteria. They were divided into a control group of 362 patients and a study group of 153 patients who received oral L-carnitine until oocyte retrieval.140 patients were matched according to maternal age, infertility duration, body mass index (BMI), day three top-quality embryos rate, by propensity score matching (PSM). The study investigated the relationship between L-carnitine treatment and *in vivo* oocyte maturation, normal fertilization, and subsequent embryo development.

**Results:**

Following PSM, initial differences in BMI and Day3 top-quality embryo rate between groups were nullified, we created two comparable cohorts with highly similar characteristics. In the subsequent cycles, the study group showed significant improvements in *in vivo* oocyte maturation rate at retrieval (p=0.002), normal *in vitro* fertilization rate (p=0.003), blastocyst formation rate (p=0.003), and usable blastocyst rate compared to controls. Although there was no significant difference in the top-quality embryo rate on Day 3, the study group showed a 10% increase in the upper quartile (55.35% vs. 66.67%). The cumulative clinical pregnancy and live birth rates showed a significant improvement (59.82% vs. 68.42%,p=0.004, 47.41% vs. 59.80%, p=0.002). Furthermore, self-control analysis revealed substantial enhancements (p<0.001) in all outcome measures following L-carnitine administration, resulting in the birth of 74 healthy neonates without congenital anomalies.

**Conclusion:**

We theorized that daily oral intake of L-carnitine before oocyte retrieval could boost oocyte quality and embryonic development, thus improving IVF outcomes. Ongoing investigations hold the potential to offer valuable insights into the applications and mechanisms underlying the therapeutic effectiveness of L-carnitine.

## 1 Introduction

Achieving fertility and ensuring the birth of healthy offspring relies on a delicate balance of various physiological processes, including endocrine, cellular, and molecular mechanisms, all of which are heavily influenced by maternal health. Metabolic disorders have emerged as significant disruptors of reproductive physiology, frequently resulting in subfertility (1). The global prevalence of these metabolic health disorders is rapidly increasing and has been solidly linked to the issue of subfertility, as noted by the World Health Organization, the United Nations Population Fund, and the Practice Committee of the American Society for Reproductive Medicine (2, 3). Reproduction, being a multifaceted process rooted in folliculogenesis, intricately depends on maternal well-being and involves complex interactions among various physiological factors, including endocrine signaling, cellular processes, and molecular pathways (4, 5). Maternal metabolic disorders can induce lipotoxic effects on developing oocytes, as shown by elevated serum non-esterified fatty acid concentrations in follicular and oviductal fluids, ultimately affecting oocyte and embryo quality (6, 7).

Mitochondria, crucial regulators of cellular metabolism, play a central role in ensuring oocyte developmental competence. Dysfunctional mitochondria under metabolic stress conditions are increasingly associated with reduced oocyte quality (8). High-fat microenvironments have been found to induce mitochondrial dysfunction in both animal models and human studies (9, 10).

L-carnitine, the sole molecule capable of transporting fatty acids into mitochondria, facilitates long-chain fatty acid transport for β-oxidation and energy production. Acting as a mitochondrial modulator, it shows promise in mitigating lipotoxic stress in oocytes by promoting fatty acid uptake and β-oxidation (11–13). However, achieving therapeutic plasma levels of L-carnitine through oral administration poses challenges due to its poor absorption and bioavailability, necessitating efficient supplementation strategies (14). In contrast to reports showing L-carnitine treatment improving embryo development *in vitro*, little is known about the effects of circulating L-carnitine or *in vivo* L-carnitine supplementation on oocyte quality (15, 16).

This study aimed to investigate the impact of oral L-carnitine administration on IVF outcomes in patients with previous treatment failure. Self-control and PSM methodologies were used to ensure reliable results, comparing outcomes before and after L-carnitine supplementation within the same individual and creating comparable groups based on the likelihood of receiving L-carnitine. The findings could significantly influence reproductive medicine, potentially offering a safe, cost-effective, and non-invasive intervention to enhance reproductive success rates and alleviate the emotional and financial burden of infertility for affected couples.

## 2 Methods

### 2.1 Study design

We included patients undergoing their second IVF cycle from January 2018 to May 2023 at the Weifang People’s Hospital Reproductive Medicine Centre. The exclusion criteria were as follows: patients with multiple IVF failures, chromosomal abnormalities, azoospermia, obvious uterine malformation, repeated implantation failure, recurrent spontaneous abortion, and severe intrauterine adhesion. Patients who changed modes of fertilization and ovarian stimulation regimens were also excluded. In total, 515 patients were ultimately included.

All included patients were randomly assigned to the study or control group using computer-generated random numbers placed in sealed, unmarked envelopes. Eligible patients who agreed to participate received an envelope, with group assignment based on whether the generated number was odd or even; this formed our historical cohort. The PSM approach was then used to correct for post-randomization differences, creating a current cohort that was balanced for baseline characteristics between the study and control groups. The ratio between the study group and the control group was set at 1:2 to facilitate a higher PSM matching rate with the expanded control group. All patients provided written informed consent. Patients, nurses, and physicians were not blinded to the assigned groups.

**Figure 1:**
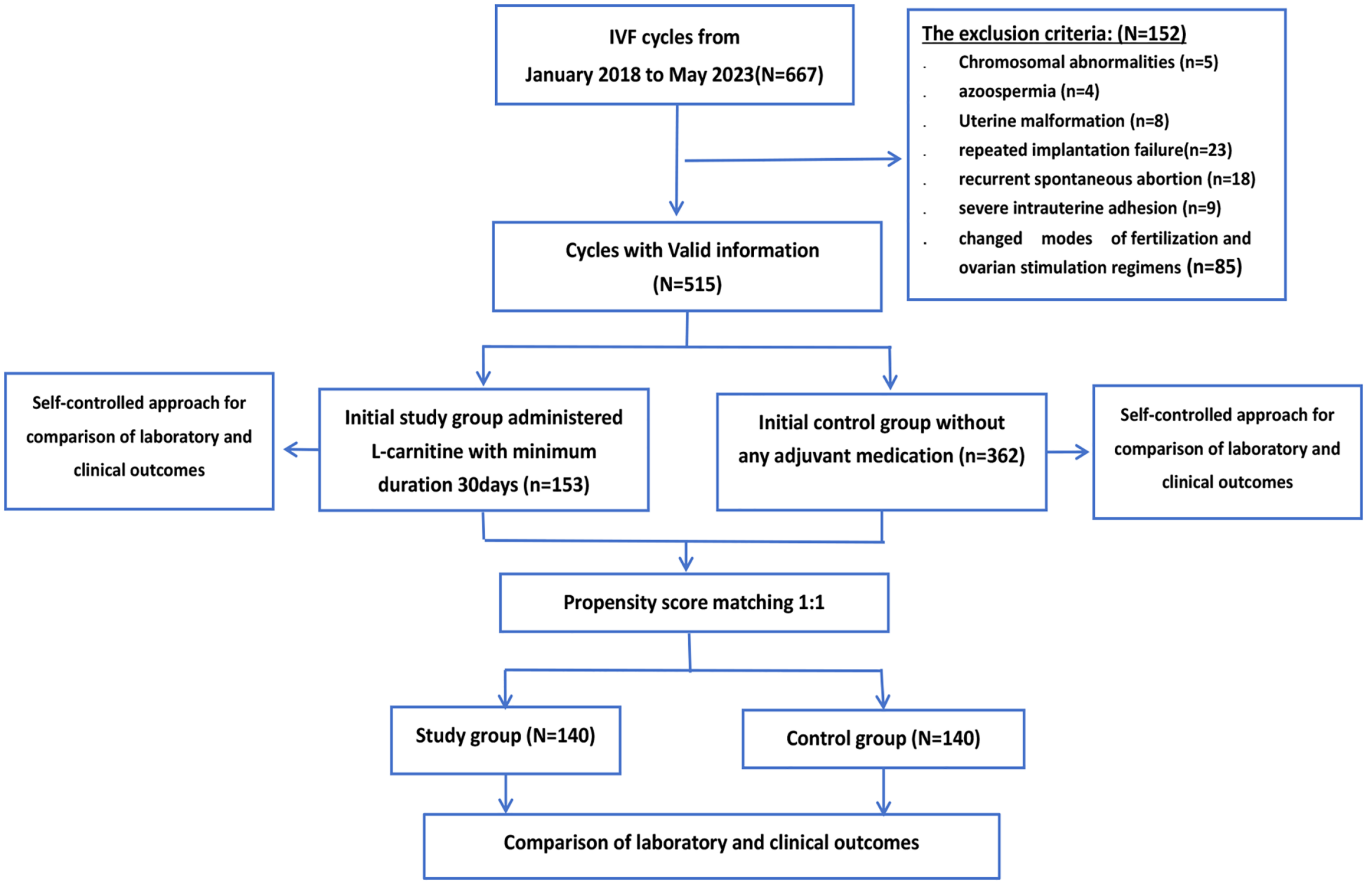
Flowchart detailing the patient inclusion and exclusion process.

The control group included 362 patients who had not undergone any adjuvant medication prior to the new IVF cycle, and 153 patients were selected as the study group. Women of all ages were included in this study, and ovarian reserve function and anti-Müllerian hormone (AMH) levels were disregarded during patient selection. The median baseline age of the study group was 35 years (IQR,31-40 years) and that of the control group was 35 years (IQR,31-40 years). There was no statistically significant difference in the median baseline levels of AMH between the study group and the control group (p=0.428). The median AMH level was 1.36 ng/mL (IQR, 0.55-2.53 ng/ml) in the study group and 1.10 ng/mL (IQR, 0.41-2.98 ng/ml) in the control group. The flowchart of the study design is shown in **Figure 1**.

The patients in the study group were administered an oral dose of 3,000 mg L-carnitine (Northeast Pharmaceutical Group, Shenyang, China) per day until the oocyte retrieval day in subsequent cycles. The average duration of oral L-carnitine administration was 44 days (30–52 days). We standardized the endpoint to the date of oocyte retrieval, but standardizing the duration was challenging due to significant individual variation in the time from when patients decided to start a new IVF cycle to the date of oocyte retrieval. The minimum duration was set as 30 days because a previous study confirmed that approximately 30 days of L-carnitine may be enough to increase fertility competence (17).

### 2.2 Ovarian stimulation

Appropriate ovarian stimulation protocols, such as gonadotropin-releasing hormone (GnRH) agonist long, GnRH antagonist, and microstimulation protocols, were selected according to the patients’ individual situation.

#### 2.2.1 GnRH agonist long protocol

In the mid-luteal phase, short-acting GnRH-a (0.1, 0.05, or 0.03 mg/day) was administered for pituitary down regulation. Initiation occurred 14–21 days after administration of GnRH agonist considering the serum levels of follicle-stimulating hormone, luteinizing hormone, and estrogen were <5 IU/L, <5 IU/L, and <50 pg/ml, respectively, and follicle diameter was ≤10 mm. Then, 100–300 IU recombinant follicle stimulating hormone was administrated depending on the patient’s age, AMH level, and antral follicle counts.

#### 2.2.2 GnRH antagonist protocol

On days 2–4 of the menstruation cycle when the sinus follicle diameter was 4–6 mm, 100–300 IU recombinant follicle-stimulating hormone was administered to initiate ovarian stimulation. If the dominant follicle was ≥12 mm or the serum levels of estradiol or luteinizing hormone were >500 pmol/l or >10 IU/L, respectively, GnRH antagonist was administered at a dose of 0.25 mg/day until the day before trigger.

#### 2.2.3 Microstimulation protocol

The microstimulation protocol is suitable for patients with poor ovarian reserve; thus, controlled ovarian stimulation was initiated for such patients on day 3 of their menstrual cycle using clomiphene or letrozole. Additionally, 150–225 IU gonadotropin was administrated.

#### 2.2.4 Trigger day

We monitored the ovarian follicle developmental progress using vaginal ultrasound scanning and estradiol levels during treatment. If the patients had at least three dominant follicles with diameters >18 mm and 50%–60% of the follicles were ≥16 mm, recombinant human chorionic gonadotropin (hCG) (250 μg) or normal hCG (6,000–10,000 IU) was injected for trigger.

### 2.3 Oocyte recovery, fertilization, and embryo culture

Transvaginal follicle aspiration was performed with ultrasound guidance under intravenous anesthesia 35–37 h after hCG injection. The collected oocytes were fertilized through conventional IVF or intracytoplasmic sperm injection at 39–40 h after hCG injection depending on the patient’s condition. All embryos were cultured using sequential culture medium (Vitrolife, Gothenburg, Sweden) in incubators at 37°C under 5% O_2_, 6% CO_2_, and 89% N_2_ conditions with high moisture until day 6.

### 2.4 Outcome measures

Number of Retrieved Oocytes: The total count of oocytes retrieved during the IVF procedure, assessed through stereomicroscopy assessment following oocyte retrieval.

Oocyte Maturation Rate: It is generally related to nuclear maturity, being defined as the proportion of oocytes at MII stage (18).

Normal fertilization: Defined as the appearance of two protonuclei and two polar bodies at 16~18h after insemination (18).

Day three (D3) top-quality embryos: They are generally defined as those originating from normally fertilized oocytes with a cell count of 7–9 on day three after fertilization, the fragmentation rate is less than 10%, and there are no multinucleated embryos (19).

Blastocyst formation rate: Defined as the proportion of 2PN zygotes (not just of cleaved zygotes) which are at the blastocyst stage at Day 5 (116 ± 2 h post-insemination) (18), We employed the Gardner system to evaluate the quality of blastocysts in terms of their expansion, inner cell mass, and trophoblast development (20).

Usable blastocyst rate: This term denotes the combined count of transferred and frozen blastocysts within a single oocyte retrieval cycle. In this study, we decided to exclude the embryo and blastocyst usable rates, as described in the Vienna Consensus. These rates were defined as the ratio of embryos (or blastocysts) suitable for transfer or cryopreservation based on the number of normally fertilized (2PN) oocytes observed on Day 1 (18). Our laboratory personalizes treatments for patients, some of whom undergo freezing on Day 3 or transfer, while the remaining embryos of the cohort are continuously cultured to blastocyst. Meanwhile, others extend embryo culture to blastocysts without Day 3 freezing or transfer. This approach introduces fluctuations in the metric due to the Day 3 freezing/transfer strategy, presenting challenges in standardizing the quality of Day 3 frozen/transfer embryos. To tackle this issue, we focused on the usable blastocyst rate indicator and introduced the concepts of maximum and minimum values. This approach aimed to mitigate the impact of embryo transfer/freezing on Day 3. The usable blastocyst rate—maximum value assumes that all embryos frozen/transfer on Day 3 can be cultured into usable blastocysts. Conversely, the usable blastocyst rate—minimum value assumes that none of the embryos frozen/transfer on Day 3 can be cultured into usable blastocysts.

Cumulative clinical pregnancy and cumulative live birth rate are defined as the delivery of at least one live birth resulting from the initial oocyte retrieval, including all fresh embryo transfers and any subsequent frozen embryo transfers, until the first clinical pregnancy/live birth delivery or until all embryos are used, whichever occurs first.

## 3 Statistical analysis

The sample size determination utilized the PASS software. Data acquisition involved results from 30 cases in both the study and control groups. Key outcome indicators included the normal fertilization rate and the usable blastocyst rate. The change was computed using a two-sided t-test at a significance level of 5%. Upon careful estimation, we determined that assigning 135 patients to both the study and control groups would yield more than 80% power to detect between-group differences.

The Data were analyzed using the Statistical Package for the Social Sciences version 27.0 (SPSS IBM Corp, Armonk, NY, USA). Measured continuous variables were shown as median (interquartile range, IQR), as none of them were subjected to normal distribution. In the preceding cycle, given to the data of the two groups were independent sample, Mann–Whitney U test was used for comparison between the two groups. Categorical variables were expressed as number (percentage) and the Chi-square test or Fisher’s exact test was selected for statistical analyses, as appropriate.

Propensity score matching (PSM) conducts up to 1:1 nearest neighbor matching with a caliper of 0.05 to balance the baseline and improve the comparability between groups. The matching items included age, infertility duration, BMI, AMH, and the day three (D3) top-quality embryos rate, which were collected from the preceding cycle, and matched patients were compared for relevant indicators in the subsequent cycle using Wilcoxon Signed-Rank test as properties of paired samples.

## 4 Results

**Figure 1** illustrates the flowchart detailing the patient inclusion and exclusion process. Prior to matching, 515 cycles meeting the inclusion criteria were considered for this study. Among them, 153 cycles involved patients who received an oral dose of 3,000 mg L-carnitine per day for a minimum duration of 30 days until the oocyte retrieval day in subsequent cycles. Following propensity score matching (PSM), 140 cycles with L-carnitine pretreatment were successfully matched with 140 routine IVF cycles. **Tables 1**–**3** present the results of the non-parametric test, comparing individual patient percentages. **Supplementary Tables S1**, **S2** include a chi-square test for grouped patients, considering the data as a whole. The consistency of our test results lends robustness to our findings.

**Table 1:** Baseline characteristics and laboratory outcomes for patients in the preceding cycle before and after PSM.

	Before PSM			After PSM		
	Control group (n = 362)	Study group (n = 153)	P-value	Control group (n =140)	Study group (n=140)	P-value
Age (years)	35 (31-40)	35 (31-40)	.332	33 (30-39)	35 (30.5-40)	.555
Infertility duration (years)	3 (1-4)	3 (2-5)	.062	3 (1-5)	3 (2-5)	.896
BMI (kg/m^2^)	23.40 (21.11-25.96)	22.40 (20.89-24.22)	.005	23.00 (20.80-25.40)	22.58 (21.02-24.28)	.375
AMH (ng/mL)	1.10 (0.41-2.98)	1.36 (0.55-2.53)	.428	1.43 (0.51-3.61)	1.41 (0.59-2.59)	.929
Total GN dose	2037 (1443-2656)	1975 (1637-2612)	.625	1850 (900-2537)	1875 (1037.5-2550)	.970
GN days	8 (7-10)	8 (7-10)	.861	8 (5-9.5)	8 (5.5-10)	.768
No. of retrieved oocytes	4 (1-9)	5 (2-9)	.327	4 (2-9)	5 (2-9)	.965
The insemination patterns						
IVF % (n)	70.44 (255)	69.28 (106)	.581	72.14 (101)	74.29 (104)	.294
ICSI % (n)	19.61 (71)	22.87 (35)		20.00 (28)	22.14 (31)	
Oocyte maturation rate (%)	90.00 (66.67-100.00)	87.50 (66.67-100.00)	.519	91.98 (62.50-100.00)	87.50 (66.67-100.00)	.996
Normal fertilization rate (%)	66.67 (33.33-100.00)	66.67 (33.33-92.58)	.437	66.67 (33.33-100.00)	66.67 (35.00-92.31)	.950
Blastocyst formation rate (%)	50.00 (0.00-100.00)	50.00 (0.00-75.00)	.206	50.00 (0.00-95.83)	50.00 (0.00-75.00)	.664
Usable blastocyst rate -the max (%)	16.67 (0.00-40.00)	16.67 (0.00-33.33)	.114	16.67 (0.00-33.33)	20.00 (0.00-33.33)	.685
Usable blastocyst rate -the min (%)	0.00 (0.00-0.00)	0.00 (0.00-0.00)	.403	0.00 (0.00-0.00)	0.00 (0.00-0.00)	.909
D3 top-quality embryos rate (%)	25.00 (0.00-60.00)	0.00 (0.00-50.00)	.018	18.33 (0.00-50.00)	14.28 (0.00-50.00)	.604

Data was described as median (IQR).

PSM, Propensity Score Matching; BMI, body mass index; AMH, anti-Müllerian hormone; GN, gonadotropin;D3,day three; IQR, interquartile ran.

**Table 2:** Comparison of laboratory and clinical outcomes in subsequent cycles after propensity score matching (PSM).

	Control group (n=140)	Study group (n = 140)	P-value
Total GN dose	2100 (1606-2793)	2125 (1656-2700)	.714
GN days	9 (7-10)	9 (7-10)	.435
No. Of retrieved oocytes	6 (2-12)	6 (3-12)	.601
Oocyte maturation rate (%)	85.71 (62.50-100.00)	100.00 (80.00-100.00)	.002
Normal fertilization rate (%)	75.00 (50.00-100.00)	80.00 (66.67-100.00)	.003
Blastocyst formation rate (%)	60.00 (0.00-88.89)	71.43 (50.00-100.00)	.003
Usable blastocyst rate -the max (%)	20.00 (0.00-40.00)	28.57 (14.46-50.00)	.038
Usable blastocyst rate -the min (%)	0.00 (0.00-11.78)	0.00 (0.00-20.00)	.019
D3top-quality embryos rate (%)	33.33 (0.00-55.35)	33.33 (0.00-66.67)	.843
Cumulative Clinical pregnancy rate (%)	59.82 (67/121)	68.42 (78/114)	.004
Cumulative live birth rate (%)	47.41 (55/116)	59.80 (61/102)	.002

Data was described as median (IQR).

GN, gonadotropin; D3,day three; IQR, interquartile range.

**Table 3:** Analysis of outcomes following control group and study group: utilizing the previous IVF cycle as a self-control.

	The control group (n = 362)		P-value	The study group (n = 153)		P-value
	Previous cycles	subsequent cycles		Previous cycles	subsequent cycles	
Total GN dose	2037 (1443-2656)	2100 (1418-2700)	.025	1975 (1637-2612)	2150 (1662-2700)	.168
GN days	8 (7-10)	8.5 (7-10)	.321	8 (7-10)	9 (7-10)	.180
No. of retrieved oocytes	4 (1-9)	5 (2-9)	<.001	5 (2-9)	6 (3-11)	<.001
Oocyte maturation rate (%)	90.00 (66.67-100.00)	100.00 (71.43-100.00)	.021	87.50 (66.67-100.00)	100.00 (80.00-100.00)	<.001
Normal fertilization rate (%)	66.67 (33.33-100.00)	66.67 (50.00-100.00)	.012	66.67 (33.33-92.58)	78.57 (65.48-100.00)	<.001
Blastocyst formation rate (%)	50.00 (0.00-100.00)	61.70 (21.67-100.00)	.089	50.00 (0.00-75.00)	71.43 (50.00-100.00)	<.001
Useable blastocyst rate -the max (%)	16.67 (0.00-40.00)	25.00 (0.00-40.71)	.213	16.67 (0.00-33.33)	30.38 (15.10-50.00)	<.001
Useable blastocyst rate -the min (%)	0.00 (0.00-0.00)	0.00 (0.00-12.50)	<.001	0.00 (0.00-0.00)	0.00 (0.00-16.67)	<.001
D3top-quality embryos rate (%)	25.00 (0.00-60.00)	33.33 (0.00-60.00)	.179	0.00 (0.00-50.00)	33.33 (0.00-66.67)	<.001
Cumulative Clinical pregnancy rate (%)	24.58 (89/362)	54.54 (168/308)	<.001	22.87 (35/153)	68.29 (84/123)	<.001
Cumulative live birth rate (%)	0.00 (0/362)	44.56 (124/278)	<.001	0.00 (0/153)	58.26 (67/115)	<.001

Data was described as median (IQR).

GN, gonadotropin; D3,day three; IQR, interquartile range.

### 4.1 Baseline characteristics and laboratory outcomes for all patients in the preceding cycle

Baseline characteristics are presented in **Table 1**, reflecting the initial cycle outcomes of 515 patients before PSM. The study group comprised 153 patients, while the control group included 362 patients. Comparatively, the L-carnitine group exhibited a lower BMI(kg/m2) (23.40(21.11-25.96) versus 22.40(20.89-24.22), P=0.005 and a reduced D3 top-quality embryo rate (%) (25.00 [0.00-60.00] versus 0.00 [0.00-50.00] P=0.018) than the control group in our non-parametric tests, as summarized in **Table 1**.

To mitigate miscellaneous bias and ensure reliable results, the PSM method was employed to adjust for differences in BMI and the rate of D3 top-quality embryos between the groups. Following 1:1 propensity score matching, **Table 1** demonstrates that all baseline characteristics were no longer statistically different between the two groups, creating two comparable cohorts with highly similar characteristics.

### 4.2 Comparison of laboratory and clinical outcomes in subsequent cycles after PSM

**Table 2** presents the outcomes of patients following the subsequent follow-up, subsequent to successful PSM. The study group exhibited significantly higher oocyte maturation rates (%) (IQR) (85.71(62.50-100.00) versus 100.00(80.00-100.00), P=0.002), normal fertilization rate (%) (IQR) (75.00(50.00-100.00) versus 80.00(66.67-100.00), p=0.002), and blastocyst formation (%) (IQR) (60.00(0.00-88.89) versus 71.43(50.00-100.00), p=0.003) compared to the control group.

Given the limited number of patients undergoing fresh cycle embryo transfers, we introduced the cumulative clinical pregnancy rate and cumulative live birth rate to strengthen our conclusions. The study group showed higher cumulative clinical pregnancy rates (68.42%) compared to the control group (59.82%, p=0.004). In the study group of 140 patients, 61 have already had live births, and 38 still have frozen embryos or ongoing pregnancies. In the control group, 55 patients have had live births, with 24 still having frozen embryos or ongoing pregnancies. The statistical difference between the two groups (p=0.002) provides strong evidence for our conclusions.

**Figure 2:**
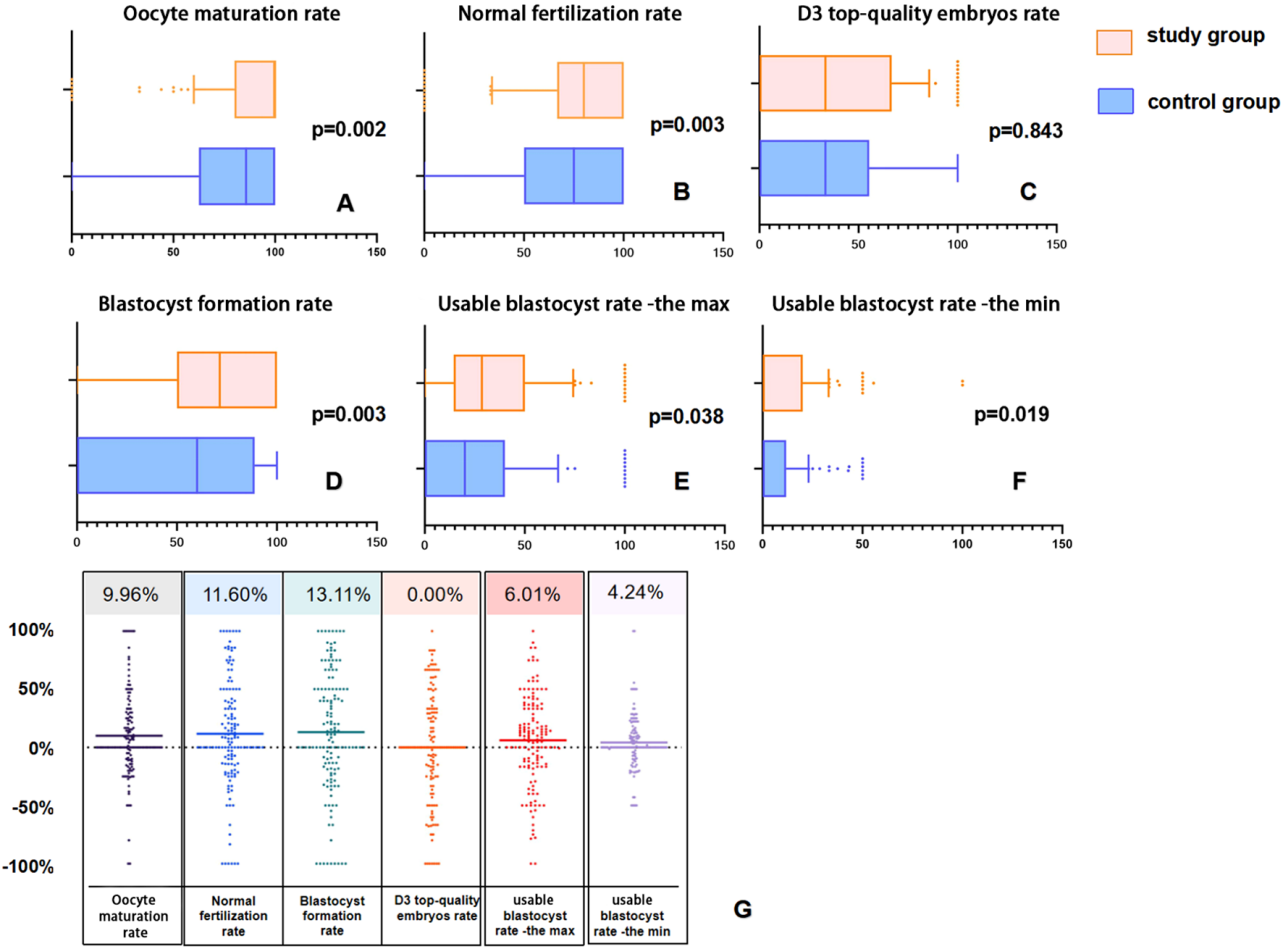
Box plots and difference scatter plots of laboratory outcomes in the matched pairs of 140 patients after PSM. In Panels **(A, B, D–F)**, compared to the control group, the distribution in the study group is noticeably shifted to the right. In Panel **(C)**, although the median of the two groups is equal, the upper quartile Q3 of the study group is noticeably shifted to the right (55.35% vs. 66.67%). In Panel **(G)**, each point represents the difference between the study group and the control group after 1:1 matching, with the upper number indicating the average of the differences.

**Figures 2A–F** display box plots visualizing the distribution of IVF laboratory outcomes for 140 patients after PSM. On the indicators of maturation rate, normal fertilization rate, blastocyst formation rate and usable blastocyst rate -the max and the min, the distribution of the study group’s data shifted significantly to the right. This trend is also seen in **Figure 2G**, which presents a scatter plot of the study group subtraction from control group.

### 4.3 Analysis of outcomes following control group and study group: utilizing the previous IVF cycle as a self-control

To assess the impact of L-carnitine, we compared outcomes from cycles following its administration with those from prior cycles, which served as the self-control group. None of the patients in this study achieved live births in their previous cycles.

The results, outlined in **Table 3**, reveal substantial improvements in laboratory and clinical outcomes. Although no significant difference in gonadotropin dosage was observed, there was a notable increase in the mean number of retrieved oocytes (IQR) (5(2-9) vs. 6(3-11), p < 0.001). Notably, seven patients who initially did not produce viable oocytes successfully retrieved oocytes following L-carnitine treatment (mean age: 37.7 years, mean AMH concentration: 0.295 ng/ml). A total of 11 oocytes were retrieved after the administration of L-carnitine, resulting in four frozen embryos containing three D3 embryos and one blastocyst.

The average oocyte maturation rate(%) (IQR) also experienced a significant rise (87.50(66.67-100.00) vs.100.00(80.00-100.00), p < 0.001), and the normal fertilization rate (%) (IQR) showed an increase (66.67(33.33-92.58) vs. 78.57(65.48-100.00), p < 0.001). A significant increase was found in the blastocyst formation rate (%) (IQR) (50.00(0.00-75.00) vs. 71.43(50.00-100.00), p < 0.001) and the usable blastocyst rate (%) (IQR)[16.67(0.00-33.33)] vs. 30.38(15.10-50.00), p < 0.001). Given the patients’ history of IVF failure, 50% initially had no D3 top-quality embryos, but this decreased to 25% after taking L-carnitine.

Before oral L-carnitine administration, 63 patients had no usable embryos (neither transferred nor frozen). These patients had an average age of 35.79 ± 5.98 years and an average of 4.69 ± 5.68 retrieved oocytes. After L-carnitine administration, only 26 patients had no usable embryos, with 17 of them having no usable embryos both before and after the treatment. These 17 patients had an average age of 38.54 ± 6.51 years and an average of 1.73 ± 1.48 retrieved oocytes. Following L-carnitine treatment, 46 patients obtained usable embryos, including 18 transferred embryos and 95 frozen embryos (59 at the Day 3 cleavage stage and 36 at the Day 5/6 blastocyst stage).

**Table 3** also showed the results of the previous cycle and the subsequent cycle for 362 controls, without any adjuvant medication. Compared to the study group, which showed statistically significant improvements in all indicators (p<.001), the control group showed improvements in the number of retrieved oocytes (p<.001), oocyte maturation rate (p=0.021), normal fertilization rate (p=0.012). However, the rates of D3 top-quality embryos, blastocyst formation, and usable blastocysts-the max did not show any improvement.

**Figure 3:**
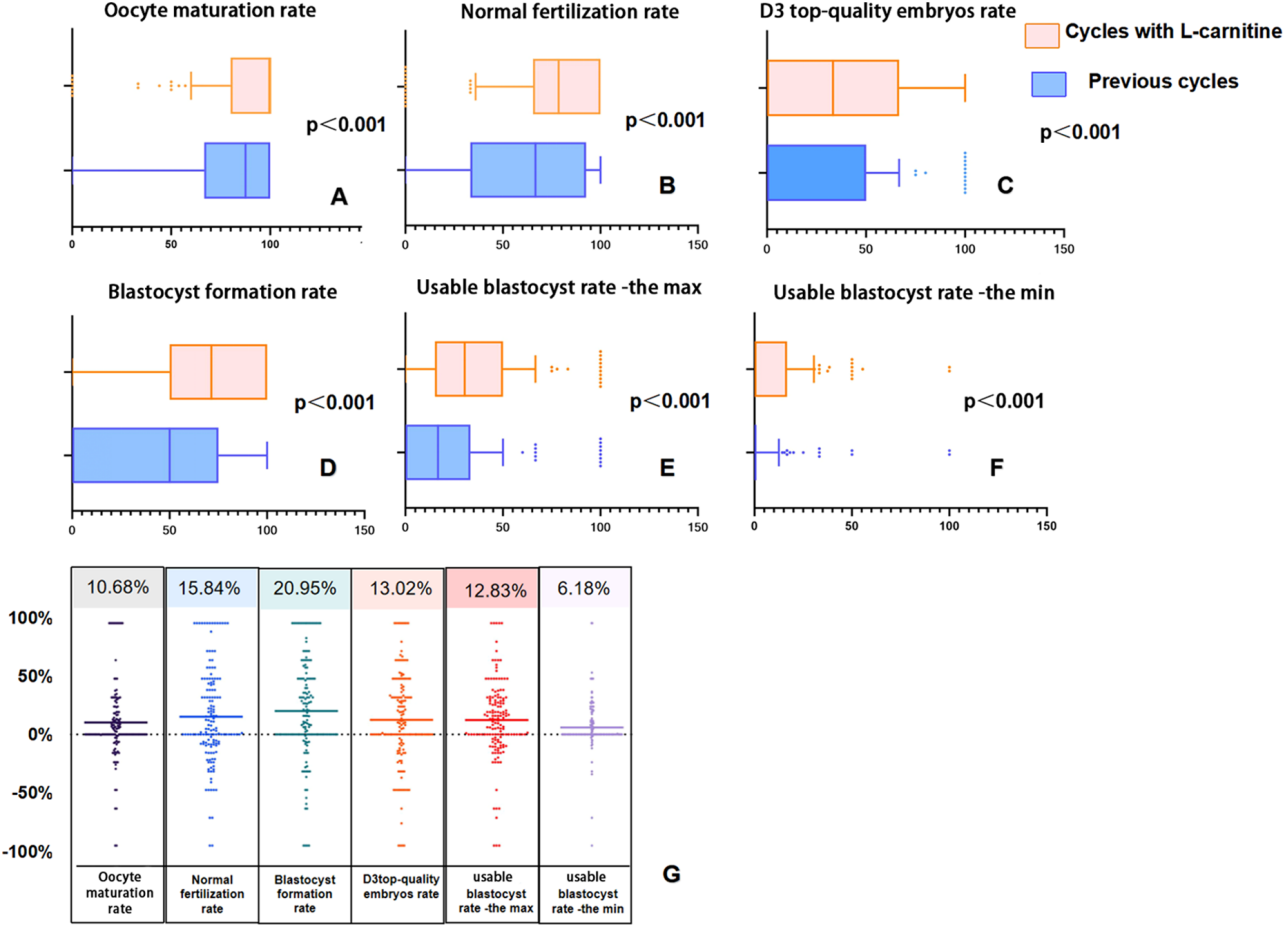
Box plots and difference scatter plots illustrating laboratory results before and after oral L-carnitine administration as self-control. In panels **(A–F)**, the overall data in the group after oral L-carnitine administration show a rightward shift. In panel **(G)**, the scatter data are densely distributed above 0% and sparse below 0%, indicating that, on average, all laboratory data have improved.

**Figure 3** presents box plots and difference scatter plots illustrating laboratory results before as self-control and after oral L-carnitine administration for 153 patients in the study group. It is noticeable from **Figures 3A–F** that there is a significant rightward shift in the distribution of laboratory indicators after administration of L-carnitine. All metrics show improvement in **Figure 3G**, with average gains ranging from 6%-20%.

### 4.4 Obstetric and neonatal outcomes after L-carnitine administration

After 67 IVF cycles following L-carnitine administration, 74 healthy babies were born, including 10 from fresh cycle transfers and 64 from frozen-thawed cycles. The mean gestational age was 38.07 ± 2.58 weeks, five of them less than 37 weeks. The mean birth height of the neonates was 50.33 ± 1.05 cm, with an average birth weight of 3,028.91 ± 662.92 g. Among them, nine had low birth weight, defined as less than 2,500 g, and three had very low birth weight, defined as less than 1,500 g. The proportion of male neonates was 43.24%, possibly influenced by the small sample size in this study (see **Table 4**).

**Table 4:** Obstetric and neonatal outcomes after L-carnitine administration.

**No. of cycles**	67
**No. of neonates**	74
**Mean gestational age (weeks)**	38.07 ± 2.58
**Preterm delivery <37 weeks**	5 (8.20%)
**Mean birth height (cm)**	50.33 ± 1.05
**Mean birth weight (g)**	3,028.91 ± 662.92
**LBW < 2,500 g**	9 (12.16%)
**VLBW < 1,500 g**	3 (4.54%)
**Congenital anomalies**	0
**Fetus gender**	Male 32 (43.24%)/female 42 (56.76%)

LBW, low birth weight; VLBW, very low birth weight.

## 5 Discussion

**Figure 4:**
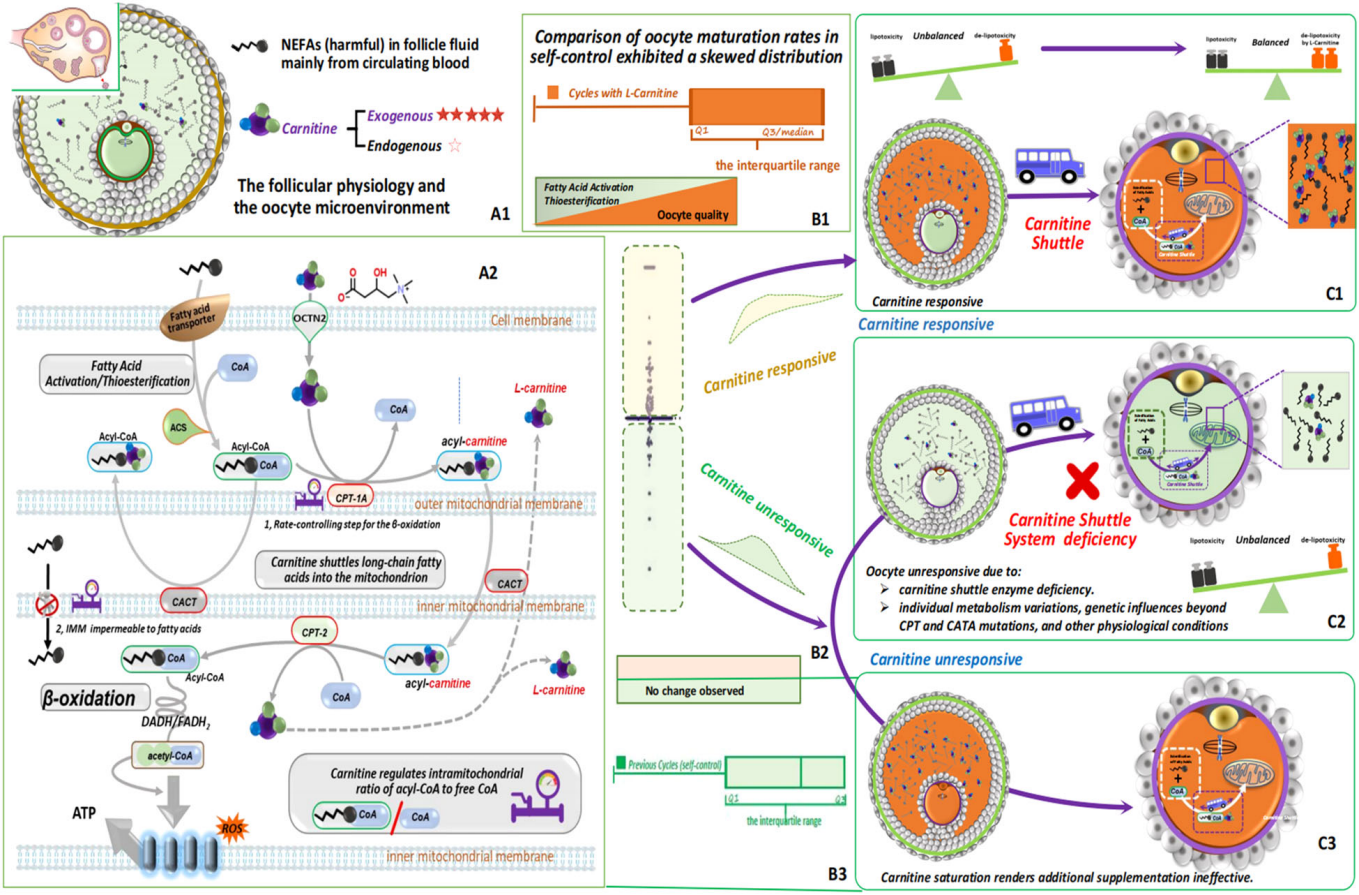
L-carnitine’s crucial role in regulating fatty acid metabolism and energy production ensures cellular lipid balance and prevents lipid toxicity. This figure illustrates its pivotal role in cellular metabolism and reproductive health. While mainly acquired from external sources, follicle cells produce minimal L-carnitine internally, indicating a potential deficiency within ovarian follicles, which can alter physiology and the microenvironment **(A1)**. L-carnitine(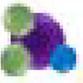) facilitates the transport of long-chain fatty acids (
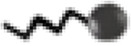
) across the mitochondrial membrane via the carnitine shuttle (
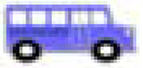
) system, ensuring efficient entry into mitochondria for β-oxidation. Key enzymes like CPT-1 and CPT-2, with CPT-1 acting as the rate-limiting step (
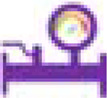
), control fatty acid β-oxidation at the mitochondrial membrane. L-carnitine also maintains the balance of acyl-CoA and free CoA within mitochondria, crucial for optimal fatty acid metabolism. Oral L-carnitine supplementation is introduced to assess its impact on follicle health and reproductive outcomes, aiming to address L-carnitine deficiency and enhance cellular metabolism and reproductive function. Fatty acids undergo conversion to fatty acyl-CoA and are transported into mitochondria via the L-carnitine shuttle for β-oxidation **(A2)**. A thorough assessment of the effects of L-carnitine supplementation is conducted by comparing the difference in *in vivo* oocyte maturation rates between the previous cycle and the L-carnitine-supplemented cycle of the study group. Data points above zero are represented by the light orange area, indicating L-carnitine responsiveness, while those below zero are represented by the light green area, indicating unresponsiveness **(B1-3, C1-3)**.

In this study, we highlight the importance of L-carnitine supplementation in enhancing reproductive outcomes, particularly among patients with varying responses to L-carnitine intake. Responsive patients demonstrate improved outcomes, emphasizing the significance of maintaining adequate L-carnitine levels for reproductive health (**Figure 4**). Conversely, unresponsive patients show minimal changes in outcomes, suggesting that factors beyond L- carnitine adequacy may contribute to their unresponsiveness. Our methodological approaches ensure a rigorous assessment of L-carnitine supplementation’s impact, yielding valuable insights into its relationship with reproductive outcomes. However, it’s essential to acknowledge the limitations of our current hypothesis.

### 5.1 Hypothesis and its limitations

Moreover, elevated levels of non-esterified fatty acids (NEFAs) in follicles can contribute to lipid toxicity, potentially leading to oxidative stress, mitochondrial dysfunction, and cellular damage, adversely affecting follicle and oocyte health (21). While L-carnitine plays a crucial role in fatty acid transport and mitochondrial function, offering a potential avenue for improving oocyte quality and *in vitro* fertilization (IVF) outcomes (22), addressing L- carnitine unresponsiveness requires personalized approaches. This may involve genetic testing, metabolic profiling, and optimization of L-carnitine supplementation strategies, including adjusting dosage, duration, or mode of administration. In cases where L-carnitine supplementation proves ineffective or contraindicated, exploring alternative interventions such as dietary modifications, lifestyle changes, or pharmacological agents targeting related pathways may be necessary to address metabolic stress and improve oocyte quality. While our study sheds light on L-carnitine responsiveness and its implications for fertility treatment, acknowledging these limitations is crucial for refining future research and clinical interventions in this domain.

### 5.2 Unlocking statistical power for IVF outcome assessment with PSM, self-control via skewed analysis

Randomized treatment assignment achieved balance in both known and unknown confounding factors between treatment groups. However, in practice, investigators could only introduce a small amount of stratification and could not balance all important variables simultaneously (23). The laboratory and clinical outcomes of IVF are influenced by a multitude of confounding factors, and the randomization of subgroups makes it difficult to achieve a completely balanced baseline. Additionally, the small sample size at our center further increased the risk of an unbalanced baseline.

Propensity score matching (PSM) was defined as the conditional probability of a subject being assigned to the treatment group given the observed covariates. Exact matching of treated and control subjects on the propensity score balances all observed covariates. This approach might provide a more informative evaluation of the efficacy of a new therapy by addressing potential confounding factors arising from imbalanced baseline features (24).

Skewed distributions associated with L-carnitine non-effective patients posed challenges for mean and standard deviation in accurately depicting data tendencies and variability. This underscores the importance of utilizing methods like the quartile-based IQR for a more accurate depiction of data spread. Unlike methods that assume data normality, the IQR avoids such assumptions, rendering it suitable for skewed datasets. By focusing on the middle 50% of the data, the IQR is resilient to outliers, in contrast to percentages, which can be skewed by outliers, potentially leading to misinterpretation.

Our findings, supported by propensity score matching between study and control groups, indicated an approximate 10% increase in oocyte maturation rate, normal fertilization rate, and blastocyst formation rate, along with a 5% rise in the usable blastocyst rate. Consistently, observations from self-control data, involving 153 patients before and after oral L-carnitine administration, revealed significant improvements in laboratory indicators, highlighting the positive impact of L-carnitine on IVF outcomes. Notably, blastocyst formation rate exhibited the highest increase at 20%, followed by the normal fertilization rate at approximately 15%. These results underscore the potential of L-carnitine to enhance reproductive interventions, offering hope for improved outcomes in IVF patients, particularly those with previous IVF failures.

### 5.3 Unraveling the impact of L-carnitine dosage and patient specificity on IVF outcomes

Our study delved into the potential of circulating L- carnitine to enhance oocyte quality *in vivo*, building upon *in vitro* observations. We theorized that daily oral intake of L-carnitine before oocyte retrieval could boost oocyte quality and embryonic development, thus improving IVF outcomes. Our investigation indeed demonstrated significant enhancements in embryo quality on days 3 and 5 following L-carnitine administration, aligning with prior research (17). This finding underscores L-carnitine’s positive influence on embryonic development, with implications for IVF success rates. In contrast to Kitano’s findings, our study revealed statistically significant improvements in rates of mature (MII) oocytes and fertilization in the study group compared to the self-control group (17).

The exclusive reliance of oocytes on L-carnitine uptake from follicular fluid emphasizes its crucial role in oocyte development. L-carnitine helps mitigate the harmful effects of non-esterified fatty acids (NEFAs) during the follicular phase and oocyte maturation, optimizing mitochondrial function and reducing lipid toxicity and oxidative stress (25).

L-carnitine is acquired both internally and externally. Research by Montjean et al. has shown minimal expression of L-carnitine synthesis pathway genes in human oocytes, and an absence of L-carnitine synthesis enzymes in cumulus cells (26). This indicates that oocytes are dependent on L-carnitine absorbed externally from follicular fluid, highlighting the importance of regulating L-carnitine in reproductive physiology. The reliance of cumulus-oocyte complexes on externally sourced L-carnitine due to the lack of synthesizing enzymes adds complexity to understanding its role in oocyte development.

The dosage of L-carnitine could explain the discrepancies between our study and Kitano’s. While Kitano administered 1000 mg daily, we used 3000 mg, emphasizing the critical role of precise dosing for consistent fertility outcomes. This difference in dosage likely contributes to the varying rates of mature oocytes and fertilization, underscoring the importance of dosage precision in achieving optimal results with L-carnitine supplementation.

Interestingly, in the control group, despite the absence of adjuvant medication, there was a statistically significant increase in the number of retrieved oocytes and the cumulative clinical pregnancy rate in subsequent cycles. This improvement may be due to clinicians adjusting the treatment protocol based on the patient’s specific characteristics during subsequent ovarian stimulations, such as modifying the dose of ovulation-promoting medication and the timing of the trigger. Additionally, lifestyle changes, such as weight loss or refraining from smoking, may also contribute to better IVF outcomes (27, 28). However, in the study group, the improvement in laboratory and clinical indicators was more pronounced, suggesting that our intervention (oral L-carnitine) had a synergistic effect. This is further supported by improved outcomes in the matched cohort comparisons within the study group relative to the control group.

However, it is important to note that studies by Sheida et al. reported varied outcomes when adding L-carnitine to antagonist ovarian stimulation protocols in IVF/ICSI cycles, particularly in women with polycystic ovarian syndrome (PCOS) (29, 30). Despite differences, these studies concluded that L-carnitine supplementation during ovulation induction in PCOS women did not significantly improve outcomes compared to control groups. Our study included a small number of PCOS patients—specifically, 5 out of 153 in our self-control group and 5 out of 140 in the PSM group—which limits our ability to investigate potential differences in this subset.

Addressing the clinical effects of L-carnitine at the intraovarian level presents challenges due to the intricate cellular and molecular aspects of PCOS, highlighting the need for further research to comprehend its clinical mechanisms across diverse patient populations, including those with PCOS, and to potentially discover new applications for ovarian dysfunctions associated with redox imbalance. Meanwhile, understanding the reasons for carnitine unresponsiveness can refine its utilization in fertility care, allowing for adjustments in factors such as dosage, duration, or administration method to enhance effectiveness for specific patient groups. This nuanced approach, which may involve exploring alternative interventions like dietary adjustments or lifestyle changes, ensures personalized treatment tailored to individual needs, thus maximizing the potential benefits for enhancing oocyte quality and fertility outcomes.

## 6 Conclusions

Our primary objective was to provide robust evidence supporting our hypothesis that daily oral consumption of L-carnitine before oocyte retrieval is beneficial. If L-carnitine supplementation proves ineffective, alternative interventions, such as dietary adjustments or targeted pharmacological agents affecting related pathways, should be explored. Recognizing these limitations is essential for refining future research and clinical strategies. Further investigations are imperative to discern L-carnitine’s clinical impacts on various patient demographics, identify factors contributing to L-carnitine unresponsiveness, and enable personalized treatment adjustments to optimize oocyte quality and fertility outcomes.

## Data Availability

The raw data supporting the conclusions of this article will be made available by the authors, without undue reservation.
