# Supplementary material for: The efficacy of orally administered L-carnitine in alleviating ovarian dysfunctions has laid the foundation for targeted in vivo use: a study employing self-control and propensity score matching
Source: Front Endocrinol (Lausanne). 2024 Sep 18;15:1440182. doi: 10.3389/fendo.2024.1440182 (PMC11445680; doi:10.3389/fendo.2024.1440182)
Supplement: Supplementary file 1 [file Table1.docx]

**TABLE 1s.** Comparison of laboratory and clinical outcomes in subsequent cycles after propensity score matching (PSM)

|  | Control group (n=140) | Study group (n = 140) | P-value |
| --- | --- | --- | --- |

| **Oocyte maturation rate (%)** | 79.56 (794/998 ) | 85.70 (899/1049) | <.001 |
| --- | --- | --- | --- |
| **Normal fertilization rate (%)** | 75.94 (603/794 ) | 79.98 (719/899 ) | .045 |
| **Blastocyst formation rate (%)** | 63.85(385/603) | 68.98 (496/719) | .048 |
| **Usable blastocyst rate -the max（%）** | 26.45(264/998) | 30.51(320/1049) | .042 |
| **Usable blastocyst rate -the min（%）** | 9.72 (97/998) | 12.68 (133/1049) | .035 |
| **D3 top-quality embryos rate (%)** | 42.95(259/603) | 39.22 (282/719 ) | .169 |
