# Supplementary material for: The efficacy of orally administered L-carnitine in alleviating ovarian dysfunctions has laid the foundation for targeted in vivo use: a study employing self-control and propensity score matching
Source: Front Endocrinol (Lausanne). 2024 Sep 18;15:1440182. doi: 10.3389/fendo.2024.1440182 (PMC11445680; doi:10.3389/fendo.2024.1440182)
Supplement: Supplementary file 2 [file Table2.docx]

**TABLE 2s**. Analysis of outcomes following L-carnitine administration: utilizing the previous IVF cycle as a self-control

|  | Previous cycles  (n = 153) | Cycles with L-carnitine  (n = 153) | P-value |
| --- | --- | --- | --- |
| **Oocyte maturation rate (%)** | 80.06 (735/918 ) | 86.10 (960/1115) | <.001 |
| **Normal fertilization rate (%)** | 67.76 (498/735) | 79.27 (761/960) | <.001 |
| **Blastocyst formation rate (%)** | 51.41(256/498) | 66.75 (508/761) | <.001 |
| **Usable blastocyst rate -the max（%）** | 18.08 (166/918 ) | 30.76 (343/1115) | <.001 |
| **Usable blastocyst rate -the min（%）** | 3.38 (31/918 ) | 11.84 (132/1115) | <.001 |
| **D3 top-quality embryos rate (%)** | 30.32 (151/498 ) | 39.03 (297/761) | .002 |
